# Decrease in Trauma Admissions with COVID-19 Pandemic

**DOI:** 10.5811/westjem.2020.5.47780

**Published:** 2020-05-22

**Authors:** Tovy H. Kamine, Adam Rembisz, Rebecca J. Barron, Carey Baldwin, Mark Kromer

**Affiliations:** * Portsmouth Regional Hospital, Department of Acute Care Surgery, Trauma, and Surgical Critical Care, Portsmouth, New Hampshire; † Portsmouth Regional Hospital, Department of Emergency Medicine, Portsmouth, New Hamsphire; ‡ University of Massachusetts, Isenberg Shool of Management, Amherst, Massachusetts

## Abstract

**Introduction:**

The COVID-19 pandemic has led to social distancing and decreased travel in the United States. The impact of these interventions on trauma and emergency general surgery patient volume has not yet been described.

**Methods:**

We compared trauma admissions and emergency general surgery (EGS) cases between February 1–April 14 from 2017–2020 in five two-week time periods. Data were compared across time periods with Poisson regression analysis.

**Results:**

There were significant decreases in overall trauma admissions (57.4% decrease, p<0.001); motor vehicle collisions (MVC) (80.5% decrease, p<0.001); and non-MVCs (45.1% decrease, p<0.001) from February–April 2020. We found no significant change in EGS cases (p = 0.70). Nor was there was a significant change in trauma cases in any other year 2017–2019.

**Conclusion:**

The COVID-19 pandemic’s burden of disease correlated with a significant decrease in trauma admissions, with MVCs experiencing a larger decrease than non-MVCs.

## INTRODUCTION

Trauma is a leading cause of death in the United States.^1^ Trauma care has improved over the last decades, although mortality and morbidity remain high. Injury prevention efforts are the leading strategy to reduce trauma-related death.^2^ Motor vehicle collisions (MVC) cause a large proportion of traumatic injuries in the US.^3^ Despite a focus on injury prevention, rates of traumatic injuries and deaths remain unchanged over the previous four decades.^1^

Coronavirus disease COVID-19 has placed many constraints on Americans. Voluntary orders were imposed in New Hampshire, which our Level II trauma center serves, to close schools on March 16, 2020, and to stay at home on March 27, 2020. These closures decreased the number of people interacting with others and out of the house in their communities.^4^ As MVCs are common mechanisms of traumatic injuries and are the most common cause of trauma at our Level II trauma center, we hypothesized that social distancing and isolation would be associated with significantly less trauma volume. Fewer people being out in their communities should result in fewer opportunities for injuries.

## METHODS

This was a retrospective analysis of previously obtained quality improvement data; institutional review board approval was not required. We reviewed trauma admissions, divided into MVC and non-MVC-related, and emergency general surgery (EGS) cases per day from February 1–April 14 of 2017, 2018, 2019, and 2020. Data were divided into five approximately equal study periods: 1) February 1–14; 2) February 15–29; 3) March 1–15; 4) March 16–31; and 5) April 1–14. The second period ended with the first confirmed COVID-19 death in the US, and the fourth period began with the closures of schools in New Hampshire. We compared trauma rates among years and time periods and years using log-linear Poisson regression models. The Poisson model was selected based on the distribution of trauma incident frequency, which is centered around low values and exhibits a right-skewed pattern with few days in which many trauma cases were recorded. These results were then followed up with pairwise Poisson rate ratio tests to further analyze the change in trauma, MVC, and non-MVC across these time periods. We used R software (R Foundation for Statistical Computing) for graphics and analysis.

## RESULTS

**Figure 1:**
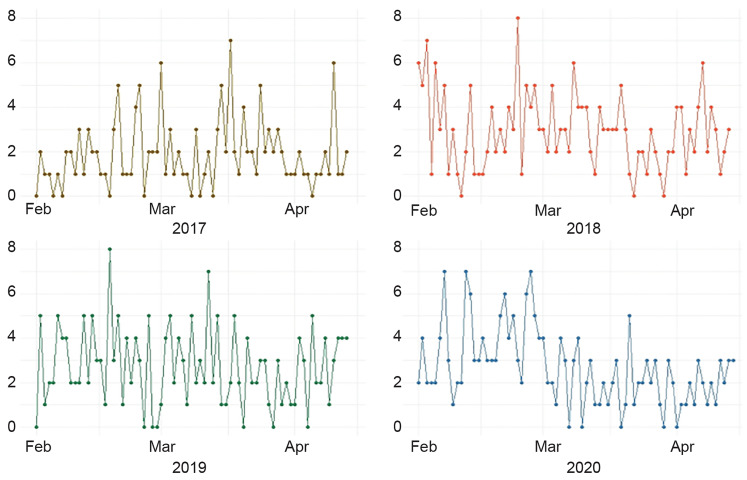
Daily trauma admissions at Portsmouth Regional Hospital from February 1–April 14, 2017–2020.

Daily trauma volume from 2017 to 2020 is displayed in Figure 1. There is a qualitative drop off visible after February 29, 2020. The overall Poisson model demonstrates a significant overall increase in trauma volume each year from 2017 to 2020 (relative risk [RR] 1.09; 95% confidence interval [CI], 1.02–1.16; p = 0.01), as well as a significant decrease from one time period to another (RR 0.93; 95% CI, 0.88–0.98, p<0.001). However, when broken down by year (Table 1), this difference between time periods is completely accounted for by the significant decreased in trauma volume in 2020 after February 29, 2020 (p <0.001). There were no significant changes across the time periods in 2017–2019. There were no significant changes in EGS cases either from 2017–2020 (RR 1.05, 95% CI, 0.92–1.20, p = 0.45) or between the individual time periods (RR 1.04, 95% CI, 0.94–1.16, p = 0.47). Similarly, when broken down by year (Table 1), there is no difference between EGS operations per day across the time periods in any individual year.

**Population Health Research Capsule**

What do we already know about this issue?

*This is the first study on the effect of COVID-19 and social distancing on trauma volume*.

What was the research question?

How did COVID-19 and social distancing affect trauma volume and emergency general surgery (EGS) volume?

What was the major finding of the study?

*There was a significant decrease in trauma volume but not EGS volume associated with the COVID-19 pandemic*.

How does this improve population health?

*Our study illustrates that population health measures enforcing social distancing also decreased trauma volume*.

When trauma admissions were broken down into MVCs and non-MVCs, there were significant decreases in both across the five time periods in 2020, with both p values <0.001 (Table 2). The percentage decrease in MVCs (80.5% decrease from peak in period 2 to trough in period 5) was larger than non-MVCs (45.1% decrease from peak in period 2 to trough in period 5).

## DISCUSSION

The COVID-19 pandemic correlated with a significant decrease in trauma volume at our Level II trauma center. Although our model showed a steady increase in trauma volume at our center year over year from 2017 to 2020, trauma volume declined significantly across the five time periods in 2020. Both MVC and non-MVC trauma were affected. As might be expected given the effects of social distancing, the percentage decrease in MVC trauma admissions (80.5%) was greater than the decrease in non-MVC trauma admissions (45.1%) across the five time periods. It is surprising that the decrease in trauma volume at our Level I trauma center started in early March, as the community mobility report suggested that overall movement in New Hampshire did not decrease until after schools were closed on March 16.^4^ We postulate that although overall movement did not decrease until March 16, people were already modifying their behavior starting in early March. Similar data showing that consumer spending, time at work, and hours worked predated state-mandated closures in many states has recently been published in the *New York Times*.^5^ As opposed to trauma volume, EGS operative volume did not change significantly across the four periods in 2020, or from year to year from 2017–2020. This is unsurprising because EGS pathologies, as opposed to trauma, are unrelated to physical distancing.

## LIMITATIONS

Although this is a small study, it is the first report on the effect of social distancing on trauma morbidity. Trauma by its nature often has much variation from month to month, so it is possible that there were other factors involved in the decrease in trauma volume in March and April 2020 compared to February. However, since there was not a significant difference between these time periods in any other year from 2017–2020, and there was a significant overall increase in trauma volume year over year, this leads credence to the conclusion that COVID-19 and the public’s fear of contracting it was responsible for the decrease. Further study is warranted as social distancing continues.

## CONCLUSION

We report a significant decrease in trauma volume starting on March 1, 2020, possibly due to the COVID-19 pandemic, without effect on EGS operative volume. As the volume appeared to decrease prior to the state-mandated social distancing, we speculate that the general public was modifying its behavior independent of government orders. It is unclear whether trauma volume from domestic violence or self-injurious behavior will increase as social distancing continues. This may impact future injury prevention efforts.\

## Figures and Tables

**Table 1:** Total trauma admissions and rmergency general surgery cases per day across five time periods from 2017 and 2020 with Poisson regression p-values.

	Year	2/1–2/14	2/15–2/29	3/1–3/15	3/16–3/31	4/1–4/14	P-value
Total trauma admissions per day	2017	1.36	2.00	1.73	2.75	1.50	0.32
	2018	3.20	3.21	3.20	2.13	2.93	0.19
	2019	2.93	3.00	3.07	2.00	2.71	0.28
	2020	3.33	4.20	2.13	1.94	1.79	**<0.001**

	Year	2/1–2/14	2/15–2/29	3/1–3/15	3/16–3/31	4/1–4/14	P-value

Emergency general surgery operations per day	2017	0.50	0.14	0.53	0.25	0.50	0.86
	2018	0.93	0.64	1.13	0.38	1.36	0.19
	2019	0.50	0.64	0.47	0.19	1.00	0.43
	2020	0.53	0.67	0.87	0.31	0.64	0.70

**Table 2:** Breakdown of 2020 trauma admissions per day into motor vehicle collisions (MVC) and non-MVCs across five time periods with Poisson regression p values.

	2/1–2/14	2/15–2/29	3/1–3/15	3/16–3/31	4/1–4/14	P-value
MVCs	0.93	1.47	0.47	0.56	0.29	**<0.001**
Non-MVCs	2.40	2.73	1.67	1.38	1.50	**<0.001**

*MVC*, motor vehicle collision.
